# Charting cancer’s course: revealing the role of diet, exercise, and the microbiome in cancer evolution and immunotherapy response

**DOI:** 10.1007/s12094-024-03595-1

**Published:** 2024-08-02

**Authors:** Ana Isabel Martin-Quesada, Maeve A. Hennessy, Ana Cardeña Gutiérrez

**Affiliations:** 1 Cell Therapy and Early Drug Development Unit, Centre Hospitalier Universitaire Vaudois (CHUV), Lausanne, Switzerland; 2 Cancer Research @UCC, University College Cork, Dublin, Ireland; 3 Hospital Universitario Nuestra Señora de Candelaria, Tenerife, Spain

**Keywords:** Physical exercise, Nutrition, Microbiome, Cancer, Immunotherapy, Response, Quality of life, Sarcopenia

## Abstract

A variety of pathophysiological mechanisms exist by which physical exercise, nutrition, and the microbiome can impact the development of cancer and the response of tumor cells to systemic anti-cancer therapy. Physical exercise positively impacts the different stages of oncological disease and may improve overall survival and quality of life, reduce treatment-associated toxicity, and improve response to immunotherapy. Nutrition impacts quality of life, and novel nutritional regimens and their role in cancer treatment and outcomes are under active investigation. Finally, the microbiome may act as a predictor of response and resistance to immunotherapy. This comprehensive review delves into the interplay between these elements and their impact on oncological outcomes, emphasizing their role in modulating the immune system and enhancing the response to immunotherapy.

The data that support the findings of this study are openly available and referenced in the bibliography section.

**Supplementary Information:**

The online version contains supplementary material available at 10.1007/s12094-024-03595-1.

## Introduction

The recognition of physical exercise as beneficial for cancer patients has grown since the 1980s [1]. Initially, small studies suggested that exercise could improve well-being and reduce fatigue during chemotherapy [1–5]. Subsequent research has confirmed that regular physical activity can help manage symptoms, improve outcomes, and possibly reduce the risk of cancer recurrence [6]. Studies also indicate that exercise modulates the tumor environment and immune response, which might enhance overall survival (OS) [7–9]. Today, incorporating exercise into cancer treatment plans is increasingly viewed as essential for improving patient health and quality of life.

Second, nutrition significantly influences the management of cancer, particularly through its impact on cachexia–anorexia syndrome, a condition marked by severe malnutrition and metabolic changes in advanced cancer patients [10]. Effective nutritional strategies, such as the Mediterranean and ketogenic diets, are being explored for their potential to improve quality of life and enhance the effectiveness of cancer treatments [11–16]. These dietary interventions, along with research into intermittent fasting, represent promising areas for improving patient outcomes in cancer care.

Alterations in the microbiota are linked to various health issues including obesity, diabetes, inflammatory diseases, and cancer [17, 18]. Dysfunctional microbiota increases intestinal permeability, allowing harmful endotoxins to promote inflammation and potentially influence cancer progression. Studies have shown that specific changes in the microbiota are associated with cancers like lung and prostate cancer [19, 20], and can affect the immune system’s response to treatments such as immune checkpoint inhibitors (ICIs) [21–25]. In addition, physical activity has been found to beneficially alter the microbiota, suggesting that exercise could enhance recovery and treatment outcomes in cancer patients.

### Exercise

#### History of physical exercise in cancer patients

The first references to the benefits of physical activity in cancer patients date back to the 1980s. In a small study of patients undergoing adjuvant chemotherapy treatment for breast cancer (BC), the implementation of physical exercise improved multiple parameters such as asthenia, physical condition, and emotional sphere [1]. In 2003, Alejandro Lucía published a review analyzing the relationship between physical exercise and asthenia in cancer patients [26]. Additional studies have attempted to demonstrate the role of exercise in reducing the risk of relapse, and some systematic reviews report a possible impact on overall survival [27].

#### In vivo and in vitro results

Tumors thrive in a hypoxic environment, often due to their rapid growth outpacing oxygen diffusion from capillaries, causing metabolic imbalance and hypoxia [28]. Aerobic exercise can alleviate this by enhancing tumor perfusion, thereby inactivating hypoxia-induced factor one (HIF-1), which increases under low oxygen conditions. HIF-1 triggers various signaling pathways, leading to angiogenesis, energy metabolism adaptation in tumor cells, and promoting metastasis and invasion, all of which contribute to tumor growth [29]. Physical exercise is suggested to regulate the tumor microenvironment by increasing oxygen pressure, thus potentially reducing tumor aggressiveness [30]. Moreover, aerobic exercise significantly improves mitochondrial content and quality, enhancing mitochondrial biogenesis and potentially delaying mitochondrial function deficiencies. It also regulates tumor vasculature and oxygenation, leading to the development of more mature, and less permeable vessels in dysfunctional tumor vasculature, thereby reducing hypoxia and improving blood flow. This contributes to normalizing the vascular network around the tumor, preventing the formation of aberrant vasculature linked to tumor progression [31].

In addition, physical exercise induces changes in mammalian target of rapamycin (mTOR) and mitogen-activated protein kinase (MAPK) pathways and muscle interleukin (IL)-6 activity [31]. The mTOR pathway promotes anabolism and energy storage and is activated by the insulin-like growth factor 1 (IGF-1) sensor [32] when nutrients are sufficient. In tumor processes, the mTOR pathway appears inappropriately hyperactivated [33], promoting cell proliferation [34].

On the other hand, the MAPK pathway maintains homeostasis of metabolism and autophagy [35, 36] through the phosphorylation of Unc-51 as kinase-1 at Ser-555 [37, 38]. The MAPK pathway acts as a tumor suppressor by countering pro-tumorigenic metabolic activities and directly triggering cell-cycle arrest in cancer cells. This pathway is modified in about 40% of all human cancers, primarily because of mutations in BRAF (approximately 10%) and its upstream activator RAS (approximately 30%) [39].

AMPK suppresses mTOR activity via two distinct pathways. The first involves the tuberous sclerosis complex 2 (TSC2). AMPK can activate TSC2, a GTPase-activating protein that teams up with TSC1, inhibiting the RAS homolog enriched in the brain (Rheb) and mTOR, ultimately preventing mTORC1 activation [40, 41]. The second pathway is based on the direct phosphorylation and inhibition of the regulatory-associated protein of mTOR, an integral part of the mTORC1 complex [42].

There is evidence that exercise could stimulate the AMPK pathway and inhibit the mTOR pathway [43].

People who do not exercise may be more prone to obesity, and in obese subjects, alterations occur in adipose tissue that lead to an increase in the release of pro-inflammatory adipokines, such as leptin or tumor necrosis factor alpha (TNF-α), which can alter homeostasis [44] and lead to a chronic inflammatory response. A prospective study showed that plasma concentrations of C-reactive protein were abnormally elevated among individuals who developed colon cancer [45]. Obesity also leads to the development of hyperinsulinemia and an increase in the levels of circulating IGF-1, a growth factor that promotes the development of many types of cancer [46].

For many years, exercise has been considered as a potential immunosuppressant. However, more recent data disputes this [47]. Exercise can enhance the immune function in cancer patients, potentially improving their response to immunotherapies by mobilizing leukocytes and reducing dysfunctional T cells. Also exercise is capable of mobilizing natural killer (NK) cells [48]. The increase in NK cell frequency is more pronounced than the increase in T and B cells.

#### Physical exercise during adjuvant treatment

Adjuvant therapies are those that are administered following surgical treatment with the aim of reducing the risk of cancer recurrence. In 2016, an important systematic review was published that included a total of 32 studies with 2626 women with localized BC [2]. Results demonstrated that physical exercise during adjuvant treatment tends to decrease fatigue and improve physical fitness with minimal impact on cancer-related quality of life (QoL) and depression. Similarly, a meta-analysis of nine randomized studies of patients with BC concluded that exercise was statistically effective in reducing fatigue [3].

In a randomized study, patients with localized BC were randomized to perform resistance exercises vs. a control group with relaxation activities [4]. In the control group, both total and physical fatigue worsened during chemotherapy, while exercise participants did not exhibit such impairments.

Another randomized study included patients with BC and colon cancer [49]. The effectiveness of a low-intensity home physical activity program *(Onco-Move)* and a combined program of supervised resistance and aerobic exercise *(OnTrack)* of moderate intensity was evaluated and this was compared to a control group. *Onco-Move* and *OnTrack* resulted in statistically significantly improved physical performance, reduced nausea and less pain. However, it is important to note that at the 6-month follow-up, most results returned to baseline levels for all three groups.

Finally, we would like to add that in a multicenter randomized study with colon cancer patients, 33 patients undergoing adjuvant treatment were randomly assigned to a group that received an 18-week supervised exercise program or usual care. The intervention group experienced significantly less physical fatigue at 18 weeks and general fatigue at 36 weeks [5].

#### Physical exercise after adjuvant treatment

In this scenario, it has been published a randomized study of physical activity carried out in previously sedentary and overweight BC patients in the first 6 months after completing adjuvant treatment [50]. The exercise intervention consisted of moderate-vigorous aerobic and resistance exercise or continuing with their previous routine. The results showed that statistically significant differences were obtained in the intervention group regarding QoL, fatigue, depression, and muscle strength.

In patients with stage I–III colon cancer in remission, the impact of physical exercise was also analyzed [51] and they were randomized into three groups to perform low- or high-intensity exercise and a control group. Over 6 months, exercise improved scores on, among others, the Pittsburgh Sleep Quality Index and the Fatigue Symptom Inventory in a dose–response manner.

#### Physical exercise and post-operative complications

Physical preparation before an intervention for lung cancer has been shown to reduce both the number of post-surgical complications and the average hospital stay [52]. This has also been demonstrated in virtual models, an interesting factor to take into account, particularly in the context of the recent SARS CoV2 pandemic [53]. In addition, mental health support, smoking cessation, and alcohol reduction are critical components, all contributing to better recovery and shorter hospital stays [54].

The studies, although heterogeneous, show that the greatest benefit is obtained from the combination of aerobic and strength exercise, with the most robust evidence demonstrated in BC and prostate cancer. It is well-documented that physical exercise programs can improve cancer-related asthenia, with resultant improvement in QoL [55].

#### Physical exercise in patients with metastatic disease

In metastatic disease, exercise has also been shown to play a role. In a study carried out with women with metastatic BC, patients who achieved cardiopulmonary function (as measured by maximal oxygen consumption VO2peak) with a VO2peak of <1.09 L/min had a median OS of 16 vs. 36 months for those who reported more than that [56].

The presence of bone metastasis can raise cause for concern, but reassuringly in a recently published review which included 17 studies, serious adverse effects were found in 4% of patients and none of these were related to bone disease [57]. However, variable results were found regarding effectiveness of physical exercise.

Another study of patients with metastatic solid cancer treated with first-line chemotherapy consisted of a 12-week intervention program of combined resistance and aerobic exercise, supervised, at home and in the hospital [58]. No adverse effects were reported and although no differences were found in lumbar muscle mass, significant differences were evident in QoL and in surrogate items such as emotional well-being and cognitive, physical, and social functions.

#### Immunotherapy, physical exercise, and cancer

In recent years, the treatment of cancer patients has been revolutionized by the arrival of immunotherapy treatment. These agents modulate the immune system to attack the tumor and have favorably impacted the prognosis of various types of tumors, with some patients achieving long-term durable responses. Thus, it is relevant to highlight that a relationship has been found with exercise practice and the response to immunotherapy treatment [7, 8]. Martin et al. [8] demonstrated that the double intervention *(exercise and nivolumab)* increased tumor necrosis and cell proliferation inhibition.

There is some data with patients already published, for example a retrospective study that investigated whether physical activity boosts the efficacy of lenvatinib plus anti-PD-1 therapy in patients with unresectable hepatocellular carcinoma. Patients were classified as active or sedentary. Active patients had significantly longer OS, progression-free survival, and higher objective response rates (ORR) than sedentary patients [9].

These results suggest that aerobic and strength training should be studied as an addition to cancer immunotherapy treatment. The immune system is likely modified with exercise, thus facilitating immunotherapy treatments to act more effectively.

#### Physical exercise and quality of life

The QoL of patients with cancer is an important factor and currently numerous studies include it as a main objective in their design. The QoL benefits of exercise are undisputed at any stage [59, 60].

In a randomized controlled trial of patients with treated locally advanced BC, patients were randomized to perform 10,000 steps per day vs. 10,000 steps per day and 12 weeks of resistance training. Results indicated significant strength improvements in participants undergoing training compared to a control group, but found no notable changes in cardiorespiratory fitness, shoulder mobility, fatigue, depression, or QoL [61].

In another randomized study, 115 patients with BC were randomly assigned to aquatic exercise, pilates or yoga. The 3 groups attended programs for 1 year and received 144 rehabilitation sessions. A significant increase in QoL was observed in participants in all groups [62].

#### Overall survival and physical exercise in cancer patients

Ultimately, OS is the most important endpoint in the evaluation of oncology patients. A study was conducted with patients with stage IV or recurrent BC [63] where a higher level of physical activity at baseline was significantly associated with a longer subsequent survival time. In addition, participating in 1 additional hour per day of moderate activity reduced the risk of subsequent mortality by 23% (HR, 0.77; 95% CI: 0.65–0.92; *P* < 0.01).

#### Mortality and physical exercise in cancer patients

In relation to mortality and physical exercise, a meta-analysis conducted by Spei et al. is worth highlighting [6]. During follow-up ranging from 3.5 to 12.7 years, there were 23.041 BC survivors, 2.522 all-cause deaths, and 841 BC deaths. Compared with women with the lowest level of physical activity, women with the highest level of activity had a lower risk of all-cause mortality (HR = 0.58, 95% CI: 0.45–0.75; 8 studies), of death from BC (HR = 0.60, 95% CI: 0.36–0.99; 5 studies) and a lower, although not significant, risk of recurrence (HR = 0.79, 95% CI: 0.60–1.05; 5 studies).

#### Relationship between intensity of physical exercise and cancer

There appears to be evidence that higher intensity of physical activity is linked to greater anti-cancer benefits [64], with an effect that could even be considered as a dose–response relationship [65].

A group of patients who survived different types of tumors were randomized to receive high-intensity (HI), moderate-intensity (MI) physical activity and a control group. It was observed that the exercise groups showed significantly greater improvements in VO2 max. Improvements in VO2 max were greater for the HI group than for MI exercise but the difference was not statistically significant. Both HI and MI groups significantly reduced general and physical fatigue, with no significant differences either. In addition, benefits were found in QoL and anxiety after HI exercise and fewer problems at work after MI exercise, compared to that with the control group [66].

#### Safety in the practice of physical activity

A review of published studies with patients who have already developed cancer concluded that physical exercise is safe in all stages of lung cancer [67].

In a meta-analysis, nine high-quality studies were included [68]. Among BC survivors, supervised aerobic exercise was statistically more effective than conventional care in improving fatigue, but with high statistical heterogeneity (*p* = 0.001; *I*(2) = 75%). No major adverse effects were reported. Courneya et al. reported five adverse events in the exercise group (lymphedema, gynecological discomfort, and viral infection), and two adverse events in the control group (foot fracture and bronchitis). Cantarero et al. reported discomfort or low-intensity pain after an exercise session in three patients.

The evidence would suggest controlled physical activity guided by trained professionals with close supervision is a reasonable approach.

#### Preparation of controlled physical exercise plans

The reported benefits of exercise, especially in breast and prostate cancer, are bringing patients closer to these controlled physical exercise programs [30, 69].

#### Physical exercise and toxicity

A randomized study in which cancer patients who had received treatment with potentially neurotoxic chemotherapies were randomly assigned to chemotherapy or chemotherapy plus exercise [70], was an individualized, moderate-intensity program of progressive walking and resistance exercises. In the intervention group, symptoms of heat/cold in hands/feet were reduced statistically significantly and numbness and tingling in a non-statistically significant manner. Exercise reduced the peripheral neuropathy more for patients who were male (*p* = 0.028), older or had BC (not statistically significant).

**Fig. 1:**
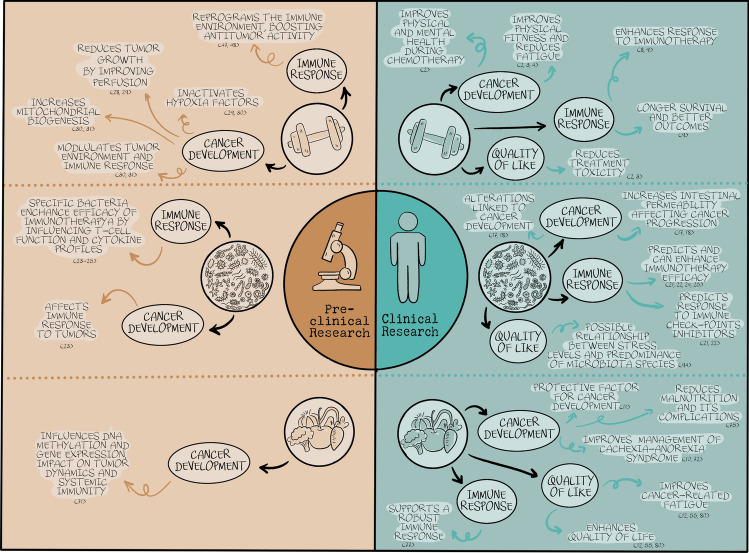
Interplay of exercise, nutrition, and the microbiome in cancer development, the response to immunotherapy, and improvements in quality of life

A figure and a table below summarize the interplay of exercise, nutrition, and the microbiome cancer development, the response to immunotherapy and improvements in quality of life (Fig. 1 and Table 1).

**Table 1:** Interplay of exercise, nutrition, and the microbiome in cancer development, the response to immunotherapy, and improvements in quality of life

Factor	Impact on cancer development	Impact on immunotherapy response	Improvements in quality of life
Exercise	**Clinical research:** – Improves physical fitness and reduces fatigue [2–4] – Improves physical and mental health during chemotherapy [2]	**Clinical research:** – Enhances response to immunotherapy [8, 9] – Associated with longer survival and better outcomes [9]	**Clinical research:** – Enhances overall survival and quality of life [2, 3] – Reduces treatment-associated toxicity [2, 3]
	**Preclinical research:** – Reduces tumor growth by improving perfusion [28, 29] – Inactivates hypoxia factors [29, 30] – Modulates tumor environment and immune response and increases mitochondrial biogenesis [30, 31]	**Preclinical research:** – Reprograms the immune environment, boosting antitumor activity [47, 48]	
Nutrition	**Clinical research:** – Improves management of cachexia–anorexia syndrome [10, 72] – Reduces malnutrition and its associated complications [75] – Protective factor for cancer development [11]	**Clinical research:** – Adequate nutrition supports a robust immune response and have a potential to improve outcomes in cancer treatment [77]	**Clinical research:** – Enhances quality of life, improves cancer-related fatigue and treatment effectiveness [12, 55, 80]
	**Preclinical research:** – Influences DNA methylation and gene expression, impact on tumor dynamics and systemic immunity [71]		
Microbiome	**Clinical research:** – Alterations linked to cancer development [17, 18] – Increased intestinal permeability affecting cancer progression [17, 18]	**Clinical research:** – Specific microbial compositions can enhance immunotherapy efficacy [21, 22, 24, 25] – Predicts response to immune checkpoint inhibitors [21, 22]	**Clinical research:** – Possible relationship between stress levels and predominance of microbiota species [93]
	**Preclinical research:** – Gut microbiota composition affects immune response to tumors [23]	**Preclinical research:** – Specific bacteria enhance efficacy of immunotherapies by influencing T cell function and cytokine profiles [23–25]	

### Diet

#### Diet and cancer: what is the interaction?

Diet also plays a fundamental role in patients who have been diagnosed with cancer. Existing evidence suggests that a diet rich in vegetables, fruits, and other plants may impact DNA methylation, histone modifications, and tumor mRNA expression [71]. Consequently, it is necessary to elucidate the impact of nutrition on epigenetics and cancer, the influence of other factors, and the optimal dietary patterns to modify epigenetics effectively.

#### Nutrition and cachexia–anorexia syndrome

Cachexia–anorexia syndrome is characterized by a state of severe malnutrition due to decreased intake and metabolic and hormonal alterations resulting from the interaction between the tumor and the host [10]. Between 50 and 80% of patients with advanced cancer develop cachexia with a subsequent 1-year mortality rate ranging from 20 to 60% [72]. Cachexia is the immediate cause of death in 20% of cancer patients. Furthermore, a weight loss >5% prior to cancer diagnosis and initiation of treatment is a predictor of early mortality regardless of stage, histology, and general condition. The European Society for Medical Oncology has recognized the importance of treating cancer-related cachexia and has published guidelines [73].

Cancer patients with weight loss receive lower doses of chemotherapy and suffer from more frequent and severe chemotherapy toxicities compared to patients with stable weight, especially stomatitis and palmar-plantar syndrome. Furthermore, they generally undergo chemotherapy for a reduced time period due to tolerance issues. This has been shown to result in significantly lower survival rates and response rates, worse QoL, and worse functional status [74].

The PREDyCES study found that malnutrition had an impact on hospital stay and costs [75]. It was found that patients initially admitted without malnutrition who were malnourished at discharge experienced a significant extension in hospital stay compared to those not malnourished at discharge (15.2 vs. 8 days), incurring an additional cost of 5,829€ per patient. Arthur et al. [76] also described longer hospitalizations for patients with cachexia and higher associated costs.

Weight loss before ICI treatment is also a predictor of worse OS, progression-free survival, and survival rates with ICI treatment [77].

#### Sarcopenia and cancer

In addition to the above, it is worth mentioning sarcopenia, which is defined as the pro-inflammatory state through which cancer patients progressively lose muscle mass and strength. In BC survivors, it was seen that sarcopenia is associated with an increased risk of overall mortality and may be associated with BC-specific mortality [78].

In the case of patients with lung cancer, the prevalence of sarcopenia can exceed 50% in some series, and its presence is related to the antitumor response obtained with onco-specific treatments, including ICIs [28, 73]. Patients with sarcopenia have been shown to have poorer survival outcomes in patients with advanced non-small-cell lung cancer receiving PD-1 blockade as compared with those without [79].

#### Nutrition and quality of life in cancer patients

Nutrition is also closely related to improved QoL for the patient. In a study mentioned before carried out within a Chinese population, 223 colorectal cancer survivors were randomly assigned to receive dietary intervention, physical or combined activity, or usual care for 12 months [55]. Participants who received dietary intervention experienced significant improvement in QoL at 12 months, as well as cancer-specific QoL scores at 12 and 24 months follow-up. Participants who received physical activity intervention alone demonstrated significant improvement in utility index and physical functioning at 6 months.

In another published review, the impact of nutrition for palliative patients is highlighted, and nutritional screening is advised to ensure appropriate dietary intervention is provided [80].

#### Mediterranean diet and cancer risk

The PREDIMED study is a multicenter randomized trial developed in Spain from 2003 to 2009. Women were randomly assigned to a Mediterranean diet supplemented with extra virgin olive oil, a Mediterranean diet supplemented with mixed nuts, or a control diet. After a median follow-up of 4.8 years, 35 confirmed cases of BC were identified. The observed rates (per 1000 person-years) were 1.1 for the Mediterranean diet with olive oil group, 1.8 for the Mediterranean diet and walnuts group, and 2.9 for the control group. The results suggested a beneficial effect of a Mediterranean diet supplemented with extra virgin olive oil in the primary prevention of BC [11].

In another study by Baguley et al. [12], patients with prostate cancer undergoing androgen deprivation therapy were randomized to receive a Mediterranean diet program with six individualized nutritional consultations vs. usual diet. It was observed that the Mediterranean diet significantly improved cancer-associated fatigue at 8 and 12 weeks. Furthermore, the intervention was shown to reduce body mass and was not associated with any adverse effects.

#### Ketogenic diet, intermittent fasting, and fasting mimicking diet in cancer patients

There is a growing interest in clinical trials exploring intermittent fasting and the ketogenic diet as interventions in cancer patients.

Several diets have been proposed for patients with cancer over the last few decades, for example “intermittent fasting”. Among the possible mechanisms that explain the potential beneficial effect of intermittent fasting is IGF-1, which may play a role in tumor growth as described above [81, 82].

On the other hand, the “ketogenic diet” is based on a limited intake of carbohydrates, which leads to a poor availability to carry out glycolysis and the consequent production of adenosine triphosphate [83]. Ketone bodies become the predominant source of energy, and thus the role of the mitochondria is essential. This, combined with the limited availability of glucose for the pentose phosphate pathway and the generation of nicotinamide adenine dinucleotide phosphate (NADPH), causes tumor cells to be subjected to oxidative stress [84]. Furthermore, the ketogenic diet and ketone bodies have been associated with a reduction in cytokines such as TNF-α, IL-1, and IL-6, with the consequent anti-inflammatory effect [85].

Preliminary studies suggest that prolonged fasting in some patients with cancer is safe and potentially capable of decreasing chemotherapy-related toxicity and tumor growth by sensitizing cells to chemotherapy [13]. The combination of prolonged courses of fasting and chemotherapy significantly improved the response to therapy in mouse xenograft models of BC, melanoma, glioma, and neuroblastoma [81].

An example is the ERGO-2 study in which 50 patients with cerebral tumors were randomized to treatment with re-irradiation combined with a standard diet vs. a ketogenic diet program in combination with intermittent fasting [14]. Twenty patients were randomized to the intervention group, all of them demonstrating good adherence and substantial decreases in leptin and insulin and an increase in uric acid. The ERGO-3 study is currently underway, which aims to establish prognostic factors and provide further insights.

In a study, 80 patients with locally advanced or metastatic BC were assigned to follow a ketogenic diet vs. a control group while undergoing chemotherapy [15]. The results showed that the intervention group received higher scores for QoL and physical activity. No significant differences in QoL or physical activity scores were observed after 12 weeks.

In another study carried out in female patients with ovarian and endometrial cancer, a ketogenic diet was not seen to negatively affect QoL [16]. The study also concluded that a ketogenic diet can improve physical function, increase energy, and decrease specific food cravings.

Regarding the “fasting mimicking diet”, which consists of periods of time of lower caloric intake, in the DIRECT study, 131 female patients with stages II and III HER2-negative BC were assigned to carry out the intervention with a low-calorie diet vs. the usual diet [86]. In the intervention group, improvements were observed in body mass index, QoL, and perception of the disease.

#### Nutrition and immunotherapy

A recent review published in 2024 explores how dietary factors such as vitamins, fatty acids, small dietary molecules, dietary patterns, and caloric restriction influence immune responses and enhance the effectiveness of anti-cancer immunotherapy. Data on this subject are increasingly being published [87].

### Microbiome

#### Alteration of the microbiota and cancer development

The microbiota is the third element to be analyzed within the holistic view of cancer, and changes in the microbiota have been analyzed in several studies. Alterations in the composition of the microbiota, particularly in the most predominant species, have been associated with obesity, diabetes, gastrointestinal inflammatory diseases and also with the development of cancer [17, 18].

An altered intestinal wall as a result of a pro-inflammatory state can increase permeability to some products produced by an altered microbiota, such as endotoxins. These circulating substances induce the expression of pro-inflammatory cytokines such as TNF-alpha, fueling the inflammation-sarcopenia cycle. Intestinal dysbiosis is strongly related to pathologies along the digestive tract, including colorectal cancer, but more recently has been described in the pathogenesis of other diseases including neurodegenerative diseases (gut–brain axis), asthma, and chronic obstructive pulmonary disease (gut–lung axis), and also with lung cancer [19].

An analysis of bronchoalveolar lavage from patients with lung cancer shows an increase in bacterial species such as TM7-3, Capnocytophaga, and Sediminibacterium, and a decrease in others such as Microbacterium and Stenotrophomonas compared to the control group [19]. An American study in which 133 patients underwent prostate biopsies demonstrated an association between the presence or absence of prostate cancer and the composition of the microbiome. Analysis revealed that patients with prostate cancer had a microbiome especially enriched in two species: *Bacteroides* and *Streptococcus* spp. [20].

#### Relationship between the microbiota and the immune system in cancer patients

There is a relationship between administered systemic anti-cancer therapies, the microbiome, and alterations in the immune system and this has been identified in a number of studies. Regarding classical treatments such as chemotherapy, Viaud et al. investigated how cyclophosphamide induced changes in the gut microbiota promoting the migration of specific bacteria to lymphoid organs, enhancing the generation of pathogenic T helper 17 cells and memory Th1 immune responses [88].

It has also been proposed that some species predominantly present in the microbiota of patients could be used as predictors of response to ICIs [21, 22]. Existing evidence suggests that specific microbial compositions can enhance the efficacy of ICI, potentially by affecting the immune system’s regulation and recognition of cancer cells. For example, Sivan et al. found a positive correlation between Bifidobacterium species enrichment and slower tumor growth in mice, noting an increase in CD8+ tumor-infiltrating T cells. Administering Bifidobacterium orally alone boosted the tumor response via CD8+ cells, mirroring anti-PD-L1 therapy effects. Combining Bifidobacterium with anti PD-L1 therapy nearly eradicated tumors in the studied mice [23].

In patients treated with anti PDL-1, treatment appears more efficacious when the hosts harbor species of bifidobacteria such as *Bifidobacterium longum*, *Collinsella aerofaciens*, and *Enterococcus faecium* [24].

On the other hand, the anti-CTLA4 therapy can allow the enrichment of *B. thetaiotaomicron* and *B. fragilis* that mediates the responses of T helper type 1 lymphocytes dependent on toll-like receptor type 4 and interleukin 12, as well as the therapeutic efficacy [25].

Identifying patients most likely to benefit from these drugs would allow resources to be directed to these individuals and other more useful therapies administered to non-responders, sparing them from unnecessary toxicities.

Finally, a meta-analysis of 107 articles representing 41,663 patients found that antibiotic use around ICI initiation was significantly associated with shorter OS and progression-free survival [89]. This negative impact on survival was consistent across cancer types, especially when antibiotics were taken shortly before or after ICI initiation.

#### Microbiota and physical activity

It is especially interesting to highlight that the microbiota is modified depending on the level of physical exercise carried out by the patient [90]. A Spanish study carried out on healthy women between 18–40 years old and homogenized in terms of their diet, divided women into three groups according to their level of physical activity. The results showed differences in the microbiota of patients according to their level of exercise, increasing the percentage of species such as *Faecalibacterium prausnitzii*, *Roseburia hominis*, and *Akkermansia muciniphila* in active patients [91], considered “healthy” bacteria within the huge spectrum of microbiota.

Another study examined the impact of gut microbiome on exercise tolerance in early-stage lung cancer patients post-surgery, incorporating an analysis of V2O metrics. The research suggested that interventions targeting the gut microbiome could potentially enhance V2O outcomes and overall recovery [92].

#### Microbiota and quality of life

Finally, there are even recent studies with patients looking at the relationship between predominant species and quality of life and stress levels [93].

## Conclusion and perspectives

In our comprehensive exploration, we have delved into the nuanced interplay between lifestyle modifications and traditional cancer therapies, revealing a multifaceted approach that extends beyond mere disease management. The integration of physical activity, personalized nutrition, and targeted microbiome adjustments has emerged as a cornerstone in augmenting the therapeutic landscape of oncology. This holistic paradigm not only seeks to improve the efficacy of standard treatments but also to empower patients with tools for self-care and resilience. Our findings underscore the critical role of such lifestyle interventions in mitigating adverse side effects, enhancing psychological wellness, and potentially curbing tumor growth through mechanisms still being unraveled.

The current evidence illustrates a significant potential for these interventions to reshape patient outcomes, suggesting a paradigm shift from a purely medicalized model of care to one that encompasses the broader determinants of health. Physical activity, with its myriad of benefits ranging from improved cardiovascular health to enhanced mood and cognitive function, stands out as a key element in patient rehabilitation and long-term recovery. Nutrition, tailored to the individual’s needs and cancer type, offers a powerful adjunct to pharmacological treatments, with the possibility of influencing tumor dynamics and systemic immunity. Meanwhile, the evolving understanding of the role of the gut microbiome not only adds a new layer of complexity, but also opens up novel therapeutic avenues that could one day result in the incorporation of microbiota-targeted interventions into standard clinical practice. Some of the results have been obtained from preclinical studies, so their confirmation in clinical trials would be advisable.

However, it should be noted that the impact of exercise on survival does not occur to the same extent across all cancer types, being breast cancer one of the cancers for which the greatest amount of evidence has been published. This evidence has perhaps been generated due to the incidence and socio-demographic factors of this type of tumor. However, we should highlight the evidence that the impact on survival obtained with moderate exercise in clinical trials in women with breast cancer can be related to the effect that this measure also has on cardiovascular mortality, as described in the Spei et al. [6] meta-analysis.

It is also worth noting in relation to the available evidence that many of the studies of physical exercise, nutrition, and microbiome in patients with cancer have small sample sizes and are heterogeneous with great variability between baseline demographic characteristics, comorbidities, tumor type, stage, treatments received, and follow-up. Furthermore, there is heterogeneity in regards to the interventions carried out and the endpoints measured, limiting comparisons and conclusions.

Large-scale, thoughtfully designed, randomized trials are warranted to safely and effectively guide recommendations. For example, it is worth considering a pilot study that seeks to identify the optimal dose of physical exercise to obtain the maximum benefit, not only in relation to the above parameters (QoL, tolerance to treatments), but also at the molecular level. Agreement regarding the most meaningful endpoints to evaluate is also necessary, for example in lung cancer, the most objective parameters to demonstrate improvement in physical capacity have been identified as peak oxygen consumption and the 6-min walk test [66].

The above data highlight the necessity of adopting a multifaceted approach in cancer care. By embracing the transformative potential of genomics and personalized medicine, it is likely that treatment strategies in the future will address both the tumor’s genetic makeup as well as the individual’s unique lifestyle factors. This strategy not only advances personalized care but also sets a new standard in oncology, prioritizing patient-centric approaches and leveraging both scientific advancements and lifestyle interventions for enhanced treatment outcomes and QoL.

### Perspectives

As we move forward, the horizon of cancer care is set to be reshaped by the integration of lifestyle interventions into the treatment paradigm. The challenge ahead lies in the translation of our findings into actionable clinical practice. To achieve this, robust multi-disciplinary research efforts, aiming to solidify the role of exercise, diet, and the microbiome within oncological treatments, are needed. Future studies must focus on delineating clear, evidence-based protocols that can be seamlessly incorporated into patient care plans. In addition, fostering patient engagement through education on the importance of lifestyle factors in their treatment journey will be vital. Embracing this holistic approach, we anticipate a shift toward more personalized, comprehensive cancer care strategies that not only aim to cure, but also enhance the patient’s QoL and long-term health. The journey ahead is promising, paving the way for an era of oncology that truly centers on the patient as a whole.

## Supplementary Information

Below is the link to the electronic supplementary material.

Supplementary file1 (DOCX 14 KB)

## Data Availability

The data sets generated during and analyzed during the current study are available from the corresponding author on reasonable request.

## References

[1] Winningham ML (1983) Effects of a bicycle ergometry program on functional capacity and feelings of control in women with breast cancer (dissertation).

[2] Furmaniak AC, Menig M, Markes MH. Exercise for women receiving adjuvant therapy for breast cancer. Cochrane Database Syst Rev. 2016;2016(9):CD005001. 10.1002/14651858.CD005001.pub3. 10.1002/14651858.CD005001.pub3 PMC6457768 27650122

[3] Lipsett A, Barrett S, Haruna F, Mustian K, O’Donovan A. The impact of exercise during adjuvant radiotherapy for breast cancer on fatigue and quality of life: a systematic review and meta-analysis. Breast. 2017;32:144–55. 10.1016/j.breast.2017.02.002. 28189100 10.1016/j.breast.2017.02.002

[4] Schmidt ME, Wiskemann J, Armbrust P, Schneeweiss A, Ulrich CM, Steindorf K. Effects of resistance exercise on fatigue and quality of life in breast cancer patients undergoing adjuvant chemotherapy: a randomized controlled trial. Int J Cancer. 2015;137(2):471–80. 10.1002/ijc.29383. 25484317 10.1002/ijc.29383

[5] Van Vulpen JK, Velthuis MJ, Bisschop CNS, Travier N, Van Den Buijs BJW, Backx FJG, Los M, Erdkamp FLG, Bloemendal HJ, Koopman M, De Roos MAJ, Verhaar MJ, Ten Bokkel-Huinink D, Van Der Wall E, Peeters PHM, May AM. Effects of an exercise program in colon cancer patients undergoing chemotherapy. Med Sci Sports Exerc. 2016;48(5):767–75. 10.1249/MSS.0000000000000855. 26694846 10.1249/MSS.0000000000000855

[6] Spei ME, Samoli E, Bravi F, La Vecchia C, Bamia C, Benetou V. Physical activity in breast cancer survivors: a systematic review and meta-analysis on overall and breast cancer survival. Breast. 2019;44:144–52. 10.1016/j.breast.2019.02.001. 30780085 10.1016/j.breast.2019.02.001

[7] Ruiz-casado A, Martín-ruiz A, Pérez LM, Provencio M, Fiuza-luces C, Lucia A. Exercise and the hallmarks of cancer. Trends Cancer. 2017;3:423–41. 10.1016/j.trecan.2017.04.007. 28718417 10.1016/j.trecan.2017.04.007

[8] Martín-ruiz A, Fiuza-luces C, Rincón-castanedo C, Fernández-moreno D, Beatriz G, Martínez-martínez E, Martín-acosta P, Coronado MJ, Franco-luzón L, Ramírez M, Provencio M, Lucia A, Provencio M, Lucia A. Benefits of exercise and immunotherapy in a murine model of human non-small-cell lung carcinoma. Exerc Immunol Rev. 2020;10:100–15. 32139351

[9] Gomes Liu X-F, Zhu X-D, Feng L-H, Li X-L, Xu B, Li K-S, et al. Physical activity improves outcomes of combined lenvatinib plus anti-PD-1 therapy in unresectable hepatocellular carcinoma: a retrospective study and mouse model. Exp Hematol Oncol. 2022;11(1):20. 35379324 10.1186/s40164-022-00275-0 PMC8978397

[10] Muliawati Y, Haroen H, Rotty LWA. Cancer anorexia—cachexia syndrome. Acta Med Indones. 2012;44(2):154–62. 22745148

[11] Toledo E, Salas-Salvado J, Donat-Vargas C, Buil-Cosiales P, Estruch R, Ros E, Corella D, Fito M, Hu FB, Aros F, Gomez-Gracia E, Romaguera D, Ortega- Calvo M, Serra-Majem L, Pinto X, Schroder H, Basora J, Sorli JV, Bullo M, Martinez-Gonzalez MA. Mediterranean diet and invasive breast cancer risk among women at high cardiovascular risk in the predimed trial a randomized clinical trial. JAMA Intern Med. 2015;175(11):1752–60. 10.1001/jamainternmed.2015.4838. 26365989 10.1001/jamainternmed.2015.4838

[12] Baguley BJ, Skinner TL, Jenkins DG, Wright ORL. Mediterranean- style dietary pattern improves cancer-related fatigue and quality of life in men with prostate cancer treated with androgen deprivation therapy: a pilot randomised control trial. Clin Nutr. 2021;40(1):245–54. 10.1016/j.clnu.2020.05.016. 32534948 10.1016/j.clnu.2020.05.016

[13] Clifton KK, Ma CX, Fontana L, Peterson LL. Intermittent fasting in the prevention and treatment of cancer. CA A Cancer J Clin. 2021;71(6):527–46. 10.3322/caac.21694. 10.3322/caac.21694 34383300

[14] Voss M, Wenger KJ, von Mettenheim N, Bojunga J, Vetter M, Diehl B, Franz K, Gerlach R, Ronellenfitsch MW, Harter PN, Hattingen E, Steinbach JP, Rödel C, Rieger J. Short-term fasting in glioma patients: analysis of diet diaries and metabolic parameters of the ERGO2 trial. Eur J Nutr. 2022;61(1):477–87. 10.1007/s00394-021-02666-1. 34487222 10.1007/s00394-021-02666-1 PMC8783850

[15] Khodabakhshi A, Seyfried TN, Kalamian M, Beheshti M, Davoodi SH. Does a ketogenic diet have beneficial effects on quality of life, physical activity or biomarkers in patients with breast cancer: a randomized controlled clinical trial. Nutr J. 2020;19(1):1–10. 10.1186/s12937-020-00596-y. 32828130 10.1186/s12937-020-00596-y PMC7443288

[16] Cohen CW, Fontaine KR, Arend RC, Soleymani T, Gower BA. Favorable effects of a ketogenic diet on physical function, perceived energy, and food cravings in women with ovarian or endometrial cancer: a randomized, controlled trial. Nutrients. 2018;10(9):1187. 10.3390/nu10091187. 30200193 10.3390/nu10091187 PMC6163837

[17] Fulbright LE, Ellermann M, Arthur JC. The microbiome and the hallmarks of cancer. PLoS Pathog. 2017;13(9):e1006480. 10.1371/journal.ppat.1006480. 28934351 10.1371/journal.ppat.1006480 PMC5608396

[18] Seelbinder B, Ni Y, Varga J, Berta J, Marfil-sa A, Weiss J, Id GP, Lohinai Z. Gut microbiome functionality might be associated with exercise tolerance and recurrence of resected early-stage lung cancer patients. PLoS ONE. 2021;16:e0259898. 10.1371/journal.pone.0259898. 34793492 10.1371/journal.pone.0259898 PMC8601557

[19] Nigro E, Perrotta F, Scialò F, D’Agnano V, Mallardo M, Bianco A, Daniele A. Food, nutrition, physical activity and microbiota: which impact on lung cancer? Int J Environ Res Public Health. 2021;18(5):2399. 10.3390/ijerph18052399. 33804536 10.3390/ijerph18052399 PMC7967729

[20] Liss MA, Robert J, Goros M, Gelfond J, Leach R, Johnson-pais T, Zhao L, Rourke E, Basler J, Ankerst D, Shah DP, Cooperberg M, Vickers A. Metabolic biosynthesis pathways identified from fecal microbiome associated with prostate cancer. Eur Urol. 2018;74:575–82. 10.1016/j.eururo.2018.06.033. 30007819 10.1016/j.eururo.2018.06.033 PMC6716160

[21] Nagasaka M, Sexton R, Alhasan R, Rahman S, Asfar AS, Sukari A. Critical reviews in oncology/hematology gut microbiome and response to checkpoint inhibitors in non-small cell lung cancer—a review. Crit Rev Oncol Hematol. 2020;145:102841. 10.1016/j.critrevonc.2019.102841. 31884204 10.1016/j.critrevonc.2019.102841

[22] Zeriouh M, Raskov H, Kvich L, Gögenur I, Bennedsen ALB. Checkpoint inhibitor responses can be regulated by the gut microbiota—a systematic review. Neoplasia. 2023;43:100923. 10.1016/j.neo.2023.100923. 37603952 10.1016/j.neo.2023.100923 PMC10465958

[23] Sivan A, Corrales L, Hubert N, Williams JB, Aquino-Michaels K, Earley ZM, Benyamin FW, Lei YM, Jabri B, Alegre ML, Chang EB, Gajewski TF. Commensal bifidobacterium promotes antitumor immunity and facilitates anti-PD-L1 efficacy. Science. 2015;350(6264):1084–9. 10.1126/science.aac4255. 26541606 10.1126/science.aac4255 PMC4873287

[24] Matson V. The commensal microbiome is associated with anti-PD-1 efficacy in metastatic melanoma patients. Science. 2017;176(3):139–48. 10.1126/science.aao3290. 10.1126/science.aao3290 PMC6707353 29302014

[25] Vétizou M, Pitt JM, Daillère R, Lepage P, Flament C, Rusakiewicz S, Routy B, Maria P, Duong CPM, Poirier-colame V, Roux A, Formenti S, Golden E, Cording S, Eberl G. Anticancer immunotherapy by CTLA-4 blockade relies on the gut microbiota. Science. 2016;350(6264):1079–84. 10.1126/science.aad1329.Anticancer. 10.1126/science.aad1329 PMC4721659 26541610

[26] Lucía A, Earnest C, Pérez M. Cancer-related fatigue: can exercise physiology assist oncologists? Lancet Oncol. 2003;4(10):616–25. 10.1016/s1470-2045(03)01221-x. 14554239 10.1016/s1470-2045(03)01221-x

[27] Mctiernan A, Friedenreich CM, Katzmarzyk PT, Powell KE, Macko R, Buchner D, Pescatello LS, Bloodgood B, Tennant B, Vaux-Bjerke A, George SM, Troiano RP, Piercy KL. Physical activity in cancer prevention and survival: a systematic review. Med Sci Sports Exerc. 2019;51(6):1252–61. 10.1249/MSS.0000000000001937. 31095082 10.1249/MSS.0000000000001937 PMC6527123

[28] Yang G, Shi R, Zhang Q. Hypoxia and oxygen-sensing signaling in gene regulation and cancer progression. Int J Mol Sci. 2020;21(21):1–24. 10.3390/ijms21218162. 10.3390/ijms21218162 PMC7663541 33142830

[29] Choudhry H, Harris AL. Advances in hypoxia-inducible factor biology. Cell Metab. 2018;27(2):281–98. 10.1016/j.cmet.2017.10.005. 29129785 10.1016/j.cmet.2017.10.005

[30] Pollán M, Barrio SC, Esteban JAC, Palmer MAS, Martín M. Exercise and cancer: a position statement from the Spanish society of medical oncology. Clin Transl Oncol. 2020;22(10):1710–29. 10.1007/s12094-020-02312-y. 32052383 10.1007/s12094-020-02312-y PMC7423809

[31] Wang Q, Zhou W. Roles and molecular mechanisms of physical exercise in cancer prevention and treatment. J Sport Health Sci. 2021;10(2):201–10. 10.1016/j.jshs.2020.07.008. 32738520 10.1016/j.jshs.2020.07.008 PMC7987556

[32] Zoncu R, Efeyan A, Sabatini DM. MTOR: from growth signal integration to cancer, diabetes and ageing. Nat Rev Mol Cell Biol. 2011;12(1):21–35. 10.1038/nrm3025. 21157483 10.1038/nrm3025 PMC3390257

[33] Zou Z, Tao T, Li H, Zhu X. MTOR signaling pathway and mTOR inhibitors in cancer: progress and challenges. Cell Biosci. 2020;10(1):1–11. 10.1186/s13578-020-00396-1. 32175074 10.1186/s13578-020-00396-1 PMC7063815

[34] Shi L, Wu Z, Miao J, Du S, Ai S, Xu E, Feng M, Song J, Guan W. Adenosine interaction with adenosine receptor A2a promotes gastric cancer metastasis by enhancing PI3K-AKT-mTOR signaling. Mol Biol Cell. 2019;30(19):2527–34. 10.1091/mbc.E19-03-0136. 31339445 10.1091/mbc.E19-03-0136 PMC6743355

[35] Fritzen AM, Madsen AB, Kleinert M, Treebak JT, Lundsgaard AM, Jensen TE, Richter EA, Wojtaszewski J, Kiens B, Frøsig C. Regulation of autophagy in human skeletal muscle: effects of exercise, exercise training and insulin stimulation. J Physiol. 2016;594(3):745–61. 10.1113/JP271405. 26614120 10.1113/JP271405 PMC5341711

[36] Meley D, Bauvy C, Houben-Weerts JHPM, Dubbelhuis PF, Helmond MTJ, Codogno P, Meijer AJ. AMP-activated protein kinase and the regulation of autophagic proteolysis. J Biol Chem. 2006;281(46):34870–9. 10.1074/jbc.M605488200. 16990266 10.1074/jbc.M605488200

[37] Egan DF, Shackelford DB, Mihaylova MM, Gelino SR, Rebecca A, Mair W, Vasquez DS, Joshi A, Gwinn DM, Asara JM, Fitzpatrick J, Dillin A, Viollet B, Hansen M, Shaw RJ. Phosphorylation of ULK1 (hATG1) by AMP-activated protein kinase connects energy sensing to mitophagy. Science. 2011;331(6016):456–61. 10.1126/science.1196371.Phosphorylation. 21205641 10.1126/science.1196371 PMC3030664

[38] Bujak AL, Crane JD, Lally JS, Ford RJ, Kang SJ, Rebalka IA, Green AE, Kemp BE, Hawke TJ, Schertzer JD, Steinberg GR. AMPK activation of muscle autophagy prevents fasting-induced hypoglycemia and myopathy during aging. Cell Metab. 2015;21(6):883–90. 10.1016/j.cmet.2015.05.016. 26039451 10.1016/j.cmet.2015.05.016 PMC5233441

[39] Santarpia L, Lippman SM, El-Naggar AK. Targeting the MAPK–RAS–RAF signaling pathway in cancer therapy. Expert Opin Ther Targets. 2012;16(1):103–19. 10.1517/14728222.2011.645805. 22239440 10.1517/14728222.2011.645805 PMC3457779

[40] Gwinn DM, Shackelford DB, Egan DF, Mihaylova MM, Mery A, Vasquez DS, Turk BE, Shaw RJ. AMPK phosphorylation of raptor mediates a metabolic checkpoint. Mol Cell. 2008;30(2):214–26. 10.1016/j.molcel.2008.03.003. 18439900 10.1016/j.molcel.2008.03.003 PMC2674027

[41] Howell JJ, Hellberg K, Turner M, Talbott G, Kolar MJ, Ross DS, Hoxhaj G, Saghatelian A, Shaw RJ, Manning BD. Metformin inhibits hepatic mTORC1 signaling via dose-dependent mechanisms involving AMPK and the TSC complex. Cell Metab. 2017;25(2):463–71. 10.1016/j.cmet.2016.12.009. 28089566 10.1016/j.cmet.2016.12.009 PMC5299044

[42] Efeyan A, Sabatini DM. mTOR and cancer: many loops in one pathway. Curr Opin Cell Biol. 2010;22(2):169–76. 10.1016/j.ceb.2009.10.007. 19945836 10.1016/j.ceb.2009.10.007 PMC2854285

[43] Richter EA, Ruderman NB. AMPK and the biochemistry of exercise: implications for human health and disease. Biochem J. 2009;418(2):261–75. 10.1042/bj20082055. 19196246 10.1042/BJ20082055 PMC2779044

[44] Unamuno X, Gómez-Ambrosi J, Rodríguez A, Becerril S, Frühbeck G, Catalán V. Adipokine dysregulation and adipose tissue inflammation in human obesity. Eur J Clin Invest. 2018;48(9):e12997. 10.1111/eci.12997. 29995306 10.1111/eci.12997

[45] Roberts DL, Dive C, Renehan AG. Biological mechanisms linking obesity and cancer risk: new perspectives. Annu Rev Med. 2010;61:301–16. 10.1146/annurev.med.080708.082713. 19824817 10.1146/annurev.med.080708.082713

[46] Erlinger TP, Platz EA, Has N, Hypoth B. C-Reactive protein and the risk of incident colorectal cancer. JAMA. 2015;291(5):585–90. 10.1001/jama.291.5.585 14762037

[47] Gustafson MP, Wheatley- CM, Rosenthal AC, Gastineau DA, Katsanis E, Johnson BD, Simpson RJ. Exercise and the immune system: taking steps to improve responses to cancer immunotherapy. J Immunother Cancer. 2021;9:e001872. 10.1136/jitc-2020-001872. 34215686 10.1136/jitc-2020-001872 PMC8256759

[48] Idonr M, Hojman P. Exercise-dependent regulation of NK cells in cancer protection. Trends Mol Med. 2016;22(7):565–77. 27262760 10.1016/j.molmed.2016.05.007

[49] Van Waart H, Stuiver MM, Van Harten WH, Geleijn E, Kieffer JM, Buffart LM, De Maaker-Berkhof M, Boven E, Schrama J, Geenen MM, Meerum Terwogt JM, Van Bochove A, Lustig V, Van Den Heiligenberg SM, Smorenburg CH, Hellendoorn-van Vreeswijk JAJH, Sonke GS, Aaronson NK. Effect of low-intensity physical activity and moderate- to high-intensity physical exercise during adjuvant chemotherapy on physical fitness, fatigue, and chemotherapy completion rates: results of the PACES randomized clinical trial. J Clin Oncol. 2015;33(17):1918–27. 10.1200/JCO.2014.59.1081. 25918291 10.1200/JCO.2014.59.1081

[50] Dieli-Conwright CM, Courneya KS, Demark-Wahnefried W, Sami N, Lee K, Sweeney FC, Stewart C, Buchanan TA, Spicer D, Tripathy D, Bernstein L, Mortimer JE. Aerobic and resistance exercise improves physical fitness, bone health, and quality of life in overweight and obese breast cancer survivors: a randomized controlled trial. Breast Cancer Res. 2018;20(1):124. 10.1186/s13058-018-1051-6. 30340503 10.1186/s13058-018-1051-6 PMC6194749

[51] Brown J. A randomized dose-response trial of aerobic exercise and health-related quality of life in colon cancer survivors. Psychooncology. 2018;27(4):1221–8. 10.1002/pon.4655. 29388275 10.1002/pon.4655 PMC5895514

[52] Cavalheri V, Granger C. Preoperative exercise training for patients with non-small cell lung cancer. Cochrane Database Syst Rev. 2017;6:CD012020. 10.1002/14651858.CD012020.pub2.www.cochranelibrary.com. 28589547 10.1002/14651858.CD012020.pub2 PMC6481477

[53] Charles C, Bardet A, Ibrahimi N, Aromatario O, Cambon L, Imbert A, Pons M, Raynard B, Sauveplane D, Pouchepadass C, Baudinet C, Lambotte O, Marabelle A, Dauchy S. Delivering adapted physical activity by videoconference to patients with fatigue under immune checkpoint inhibitors : lessons learned from the PACTIMe-FEAS feasibility study. J Telemed Telecare. 2021;29:716–24. 10.1177/1357633X211021743. 34137641 10.1177/1357633X211021743

[54] Shakya P, Poudel S. Prehabilitation in patients before major surgery: a review article. J Nepal Med Assoc. 2022;60(254):909–15. 10.31729/jnma.7545 PMC9924929 36705159

[55] Ho M, Ho JWC, Fong DYT, Lee CF, Macfarlane DJ, Cerin E, Lee AM, Leung S, Chan WYY, Leung IPF, Lam SHS, Chu N, Taylor AJ, Cheng KK. Effects of dietary and physical activity interventions on generic and cancer-specific health-related quality of life, anxiety, and depression in colorectal cancer survivors: a randomized controlled trial. J Cancer Surviv. 2020;14(4):424–33. 10.1007/s11764-020-00864-0. 32072434 10.1007/s11764-020-00864-0 PMC7360640

[56] Jones LW, Courneya KS, Mackey JR, Muss HB, Pituskin EN, Scott JM, Hornsby WE, Coan AD, Herndon JE, Douglas PS, Haykowsky M. Cardiopulmonary function and age-related decline across the breast cancer: survivorship continuum. J Clin Oncol. 2012;30(20):2530–7. 10.1200/JCO.2011.39.9014. 22614980 10.1200/JCO.2011.39.9014 PMC3397786

[57] Weller S, Hart NH, Bolam KA, Mansfield S, Santa Mina D, Winters-Stone KM, Campbell A, Rosenberger F, Wiskemann J, Quist M, Cormie P, Goulart J, Campbell KL. Exercise for individuals with bone metastases: a systematic review. Crit Rev Oncol Hematol. 2021;166:103433. 10.1016/j.critrevonc.2021.103433. 34358650 10.1016/j.critrevonc.2021.103433

[58] Park JH, Park KD, Kim JH, Kim YS, Kim EY, Ahn HK, Park I, Sym SJ. Resistance and aerobic exercise intervention during chemotherapy in patients with metastatic cancer: a pilot study in South Korea. Ann Palliat Med. 2021;10(10):10236–43. 10.21037/apm-21-1432. 34498475 10.21037/apm-21-1432

[59] Lafaro KJ, Raz DJ, Kim JY, Hite S, Ruel N, Varatkar G, Erhunmwunsee L, Melstrom L, Lee B, Singh G, Fong Y, Sun V. Pilot study of a telehealth perioperative physical activity intervention for older adults with cancer and their caregivers. Support Care Cancer. 2020;28(8):3867–76. 10.1007/s00520-019-05230-0. 31845007 10.1007/s00520-019-05230-0 PMC8805142

[60] Quist M, Langer SW, Lillelund C, Winther L, Laursen JH, Christensen KB, Rørth M, Adamsen L. Lung cancer effects of an exercise intervention for patients with advanced inoperable lung cancer undergoing chemotherapy: a randomized clinical trial. Lung Cancer. 2020;145:76–82. 10.1016/j.lungcan.2020.05.003. 32416432 10.1016/j.lungcan.2020.05.003

[61] Soriano-Maldonado A, Díez-Fernández DM, Esteban-Simón A, Rodríguez-Pérez MA, Artés-Rodríguez E, Casimiro-Artés MA, Moreno-Martos H, Toro-de-Federico A, Hachem-Salas N, Bartholdy C, Henriksen M, Casimiro-Andújar AJ. Effects of a 12-week supervised resistance training program, combined with home-based physical activity, on physical fitness and quality of life in female breast cancer survivors: the EFICAN randomized controlled trial. J Cancer Surviv. 2023;17(5):1371–85. 10.1007/s11764-022-01192-1. 35314958 10.1007/s11764-022-01192-1 PMC10442259

[62] Odynets T, Briskin Y, Todorova V. Effects of different exercise interventions on quality of life in breast cancer patients: a randomized controlled trial. Integr Cancer Ther. 2019;18. 10.1177/1534735419880598. 10.1177/1534735419880598 PMC6801883 31625419

[63] Palesh O, Kamen C, Sharp S, Golden A, Neri E, Spiegel D, Koopman C. Physical activity and survival in women with advanced breast cancer. Cancer Nurs. 2018;41(4):E31–8. 10.1097/NCC.0000000000000525. 28727578 10.1097/NCC.0000000000000525 PMC5775062

[64] De Boer MC, Wörner EA, Verlaan D, van Leeuwen PAM. The mechanisms and effects of physical activity on breast cancer. Clin Breast Cancer. 2017;17(4):272–8. 10.1016/j.clbc.2017.01.006. 28233686 10.1016/j.clbc.2017.01.006

[65] Friedenreich CM, Cust AE. Physical activity and breast cancer risk: impact of timing, type and dose of activity and population subgroup effects. Br J Sports Med. 2008;42(8):636–47. 10.1136/bjsm.2006.029132. 18487249 10.1136/bjsm.2006.029132

[66] Kampshoff CS, Chinapaw MJM, Brug J, Twisk JWR, Schep G, Nijziel MR, van Mechelen W, Buffart LM. Randomized controlled trial of the effects of high intensity and low-to-moderate intensity exercise on physical fitness and fatigue in cancer survivors: results of the resistance and endurance exercise after chemotherapy (REACT) study. BMC Med. 2015;13(1):275. 10.1186/s12916-015-0513-2. 26515383 10.1186/s12916-015-0513-2 PMC4625937

[67] Singh B, Spence R, Steele ML, Hayes S, Toohey K. Exercise for individuals with lung cancer: a systematic review and meta-analysis of adverse events, feasibility, and effectiveness. Semin Oncol Nurs. 2020;36(5):151076. 10.1016/j.soncn.2020.151076. 33008682 10.1016/j.soncn.2020.151076

[68] Meneses-Echávez JF, González-Jiménez E, Ramírez-Vélez R. Effects of supervised exercise on cancer-related fatigue in breast cancer survivors: a systematic review and meta-analysis. BMC Cancer. 2015;15(1):1–13. 10.1186/s12885-015-1069-4. 25885168 10.1186/s12885-015-1069-4 PMC4364505

[69] Correia IR, Cardoso V, Cargaleiro C, Magalhães JP, Hetherington-Rauth M, Rosa GB, et al. Effects of home-based exercise programs on physical fitness in cancer patients undergoing active treatment: a systematic review and meta-analysis of randomized controlled trials. J Sci Med Sport. 2023;26(4–5):222–31. 37002132 10.1016/j.jsams.2023.03.009

[70] Kleckner IR, Kamen C, Gewandter JS, Mohile NA, Heckler CE, Culakova E, Fung C, Janelsins MC, Asare M, Lin PJ, Reddy PS, Giguere J, Berenberg J, Kesler SR, Mustian KM. Effects of exercise during chemotherapy on chemotherapy-induced peripheral neuropathy: a multicenter, randomized controlled trial. Support Care Cancer. 2018;26(4):1019–28. 10.1007/s00520-017-4013-0. 29243164 10.1007/s00520-017-4013-0 PMC5823751

[71] Supic G, Jagodic M, Magic Z. Epigenetics: a new link between nutrition and cancer. Nutr Cancer. 2013;65(6):781–92. 10.1080/01635581.2013.805794. 23909721 10.1080/01635581.2013.805794

[72] Von Haehling S, Anker SD. Prevalence, incidence and clinical impact of cachexia: facts and numbers—update 2014. J Cachexia Sarcopenia Muscle. 2014;5(4):261–3. 10.1007/s13539-014-0164-8. 25384990 10.1007/s13539-014-0164-8 PMC4248411

[73] Arends J, Strasser F, Gonella S, Solheim TS, Madeddu C, Ravasco P, Buonaccorso L, de van der Schueren MAE, Baldwin C, Chasen M, Ripamonti CI. Cancer cachexia in adult patients: ESMO clinical practice guidelines☆. ESMO Open. 2021;6(3):100092. 10.1016/j.esmoop.2021.100092. 34144781 10.1016/j.esmoop.2021.100092 PMC8233663

[74] Andreyev HJN, Norman AR, Oates J, Cunningham D. Why do patients with weight loss have a worse outcome when undergoing chemotherapy for gastrointestinal malignancies? Eur J Cancer. 1998;34(4):503–9. 10.1016/S0959-8049(97)10090-9. 9713300 10.1016/s0959-8049(97)10090-9

[75] Álvarez-Hernández J, Planas Vila M, León-Sanz M, García de Lorenzo A, Celaya-Pérez S, García-Lorda P, Araujo K, Sarto Guerri B, PREDyCES researchers. Prevalence and costs of malnutrition in hospitalized patients; the PREDyCES study. Nutr Hosp. 2012;27(4):1049–59. 10.3305/nh.2012.27.4.5986. 23165541 10.3305/nh.2012.27.4.5986

[76] Arthur ST, Van Doren BA, Roy D, Noone JM, Zacherle E, Blanchette CM. Cachexia among US cancer patients. J Med Econ. 2016;19(9):874–80. 10.1080/13696998.2016.1181640. 27100202 10.1080/13696998.2016.1181640

[77] Johannet P, Sawyers A, Qian Y, Kozloff S, Gulati N, Donnelly D, et al. Baseline prognostic nutritional index and changes in pretreatment body mass index associate with immunotherapy response in patients with advanced cancer. J Immunother Cancer. 2020;8(2):e001674. 10.1136/jitc-2020-001674. 33219093 10.1136/jitc-2020-001674 PMC7682457

[78] Villaseñor A, Ballard-Barbash R, Baumgartner K, Baumgartner R, Bernstein L, McTiernan A, Neuhouser ML. Prevalence and prognostic effect of sarcopenia in breast cancer survivors: the HEAL study. J Cancer Surviv. 2012;6(4):398–406. 10.1007/s11764-012-0234-x. 23054848 10.1007/s11764-012-0234-x PMC3747827

[79] Shiroyama T, Nagatomo I, Koyama S, Hirata H, Nishida S, Miyake K, Fukushima K, Shirai Y, Mitsui Y, Takata S, Masuhiro K, Yaga M, Iwahori K, Takeda Y, Kida H, Kumanogoh A. Impact of sarcopenia in patients with advanced non-small cell lung cancer treated with PD-1 inhibitors: a preliminary retrospective study. Sci Rep. 2019;9(1):1–7. 10.1038/s41598-019-39120-6. 30792455 10.1038/s41598-019-39120-6 PMC6385253

[80] Marín Caro MM, Laviano A, Pichard C. Impact of nutrition on quality of life during cancer. Curr Opin Clin Nutr Metab Care. 2007;10(4):480–7. 10.1097/MCO.0b013e3281e2c983. 17563467 10.1097/MCO.0b013e3281e2c983

[81] Lee C, Raffaghello L, Brandhorst S, Safdie FM, Bianchi G, Martin-Montalvo A, Pistoia V, Wei M, Hwang S, Merlino A, Emionite L, de Cabo R, Longo VD. Fasting cycles retard growth of tumors and sensitize a range of cancer cell types to chemotherapy. Sci Transl Med. 2012;4(124):124ra27. 10.1126/scitranslmed.3003293. 22323820 10.1126/scitranslmed.3003293 PMC3608686

[82] Salvadori G, Mirisola MG, Longo VD. Intermittent and periodic fasting, hormones, and cancer prevention. Cancers. 2021;13(18):1–20. 10.3390/cancers13184587. 10.3390/cancers13184587 PMC8472354 34572814

[83] Mundi MS, Mohamed Elfadil O, Patel I, Patel J, Hurt RT. Ketogenic diet and cancer: fad or fabulous? J Parenter Enter Nutr. 2021;45:26–32. 10.1002/jpen.2226. 10.1002/jpen.2226 34897736

[84] Allen BG, Bhatia SK, Anderson CM, Eichenberger-Gilmore JM, Sibenaller ZA, Mapuskar KA, Schoenfeld JD, Buatti JM, Spitz DR, Fath MA. Ketogenic diets as an adjuvant cancer therapy: history and potential mechanism. Redox Biol. 2014;2(1):963–70. 10.1016/j.redox.2014.08.002. 25460731 10.1016/j.redox.2014.08.002 PMC4215472

[85] Plotti F, Terranova C, Luvero D, Bartolone M, Messina G, Feole L, Cianci S, Scaletta G, Marchetti C, Di Donato V, Fagotti A, Scambia G, Benedetti Panici P, Angioli R. Diet and chemotherapy: the effects of fasting and ketogenic diet on cancer treatment. Chemotherapy. 2020;65(3–4):77–84. 10.1159/000510839. 33197913 10.1159/000510839

[86] Lugtenberg RT, de Groot S, Kaptein AA, Fischer MJ, Kranenbarg EM, Carpentier MD, Cohen D, de Graaf H, Heijns JB, Portielje JEA, van de Wouw AJ, Imholz ALT, Kessels LW, Vrijaldenhoven S, Baars A, Fiocco M, van der Hoeven JJM, Gelderblom H, Longo VD, Pijl H, Dutch Breast Cancer Research Group (BOOG). Quality of life and illness perceptions in patients with breast cancer using a fasting mimicking diet as an adjunct to neoadjuvant chemotherapy in the phase 2 DIRECT (BOOG 2013–14) trial. Breast Cancer Res Treat. 2021;185(3):741–58. 10.1007/s10549-020-05991-x. 33179154 10.1007/s10549-020-05991-x PMC7921018

[87] Golonko A, Pienkowski T, Swislocka R, Orzechowska S, Marszalek K, Szczerbinski L, et al. Dietary factors and their influence on immunotherapy strategies in oncology: a comprehensive review. Cell Death Dis. 2024;15(4). 10.1038/s41419-024-06641-6 PMC11004013 38594256

[88] Viaud S, Saccheri F, Mignot G, Yamazaki T, Hannani D, Enot DP, Pfirschke C, Engblom C, Pittet J, Schlitzer A, Ginhoux F, Apetoh L, Chachaty E. The intestinal microbiota modulates. Science. 2013;342(6161):971–6. 10.1126/science.1240537. 24264990 10.1126/science.1240537 PMC4048947

[89] Crespin A, Le Bescop C, de Gunzburg J, Vitry F, Zalcman G, Cervesi J, et al. A systematic review and meta-analysis evaluating the impact of antibiotic use on the clinical outcomes of cancer patients treated with immune checkpoint inhibitors. Front Oncol. 2023;13:1075593. 10.3389/fonc.2023.1075593. 36937417 10.3389/fonc.2023.1075593 PMC10019357

[90] Perez L, Ramı JD, Aya V, Flo A. Association between physical activity and changes in intestinal microbiota composition: a systematic review. PLoS ONE. 2021;16:e0247039. 10.1371/journal.pone.0247039. 33630874 10.1371/journal.pone.0247039 PMC7906424

[91] Pe J, Bressa C, Montalvo-lominchar MG, Mate JL, Pe M. Differences in gut microbiota profile between women with active lifestyle and sedentary women. PLoS ONE. 2017;12:e0171352. 10.1371/journal.pone.0171352. 28187199 10.1371/journal.pone.0171352 PMC5302835

[92] Marfil-Sánchez A, Seelbinder B, Ni Y, Varga J, Berta J, Hollosi V, Dome B, Megyesfalvi Z, Dulka E, Galffy G, Weiss GJ, Panagiotou G, Lohinai Z. Gut microbiome functionality might be associated with exercise tolerance and recurrence of resected early-stage lung cancer patients. PLoS ONE. 2021;16(11):e0259898. 10.1371/journal.pone.0259898. 34793492 10.1371/journal.pone.0259898 PMC8601557

[93] Lee C-C, Yang H-W, Liu C-J, Lee F, Ko W-C, Chang Y-C, et al. Unraveling the connections between gut microbiota, stress, and quality of life for holistic care in newly diagnosed breast cancer patients. Sci Rep. 2023;13(1):17916. 37864098 10.1038/s41598-023-45123-1 PMC10589294

