# Supplementary material for: Charting cancer’s course: revealing the role of diet, exercise, and the microbiome in cancer evolution and immunotherapy response
Source: Clin Transl Oncol. 2024 Aug 2;27(2):473–85. doi: 10.1007/s12094-024-03595-1 (PMC11782318; doi:10.1007/s12094-024-03595-1)
Supplement: Supplementary file 1 — Supplementary file1 (DOCX 14 KB) [file 12094_2024_3595_MOESM1_ESM.docx]

**Supplementary appendix: Summary of the searches carried out for the review.**

| **Terms searched** | **Filters** | **Search Period** | **Results Yielded** |
| --- | --- | --- | --- |
| "Diet" OR "nutrition" OR "exercise" OR "physical activity" AND "cancer" OR "Neoplasms" | Randomized clinical trials | Last 5 Years | 1.351 studies |
| “Diet” OR “Nutrition” AND “cancer” or “Neoplasms” | Randomized clinical trials | Last 5 Years | 693 studies |
| "Exercise" OR "physical activity" AND "cancer" OR "Neoplasms" | Randomized clinical trials  Spanish  English | Last 5 Years | 873 studies |
| "Anaerobic glycolysis" AND “cancer” | No filters | Last 10 Years | 12.094 studies |
| "Insulin" OR "mTOR" OR "AMPK" AND "cancer" OR "Neoplasms" | Systematic reviews and meta-analyses | Last 10 years | 50 studies |
| "Insulin" AND "mTOR pathway" | No filters | No limit | 932 studies |
| "Exercise" AND "Immunotherapy" | No filters | No limit | 399 studies |
